# Telecoaching Improves Positive Pressure Ventilation Performance During Simulated Neonatal Resuscitations

**DOI:** 10.1089/tmr.2021.0049

**Published:** 2022-03-07

**Authors:** Mark Castera, Megan M. Gray, Carri Gest, Patrick Motz, Taylor Sawyer, Rachel Umoren

**Affiliations:** ^1^ Department of Pediatrics, University of Washington School of Medicine, Seattle, Washington, USA.; ^2^ Department of Pediatrics, Seattle Children's Hospital, Seattle, Washington, USA.; ^3^ Department of Neonatology, University of Washington Medical Center, Seattle, Washington, USA.; ^4^ Department of Neonatology, Roseville Medical Center, Roseville, California, USA.

**Keywords:** telemedicine, resuscitation, simulation, neonatal, ventilation, education

## Abstract

**Introduction:**

Positive pressure ventilation (PPV) is a critical skill for neonatal resuscitation. We hypothesized that telecoaching would improve PPV performance in neonatal providers during simulated neonatal resuscitations.

**Setting:**

Level IV neonatal intensive care unit (NICU).

**Methods:**

This prospective crossover study included 14 experienced NICU nurses and respiratory therapists who performed PPV on a mannequin that recorded parameters of ventilation efficiency. Participants were randomized to practice independently (control) or with live feedback from a remote facilitator through audiovisual connection (intervention) and then switched to the opposite group. Participants' mask leak percentage, ventilation rates, and pressure delivery were analyzed.

**Results:**

The primary outcome of mask leak percentage was significantly increased in the telecoaching group (19% [interquartile range {IQR} 14–59.25] vs. 100% [IQR 88–100] leak, *p* = 0.0001). The secondary outcome of peak inspiratory pressure (PIP) delivery was also increased (median 27.6 [IQR 23.5–34.7] vs. 23.3 [IQR 19.1–32.8] cmH_2_O, *p* < 0.001). Differences in ventilation rates were not statistically significant (55 vs. 58 breaths/min, *p* = 0.51).

**Conclusion:**

Participants demonstrated better PPV performance during telecoaching with less mask leak. The intervention group also had higher measured peak inspiratory pressures. Telecoaching may be a feasible method to provide real-time feedback to health care providers during simulated neonatal resuscitations.

**Hypothesis:**

Neonatal providers who receive telecoaching during simulated resuscitations will perform PPV more effectively than those who do not receive telecoaching.

## Introduction

Approximately 10% of neonates require positive pressure ventilation (PPV) at birth.^1^ Proper PPV for neonates with respiratory depression reduces serious morbidity and mortality from perinatal asphyxia.^2^ Opportunities to perform neonatal resuscitation program (NRP) skills are relatively rare for many providers, especially those practicing at lower-volume delivery hospitals.^3^

Simulation education through standardized curricula such as the NRP and Helping Babies Breathe (HBB) plays a crucial role in establishing these skills in newborn care providers.^4^ Unfortunately, delivering simulation education can be challenging in low-resource or remote settings, and providers' skills often deteriorate within a few months of training.^5–7^ As frequent in-person refresher training is time and resource-intensive, health care workers may benefit from real-time virtual support to provide optimal care in clinical settings.^8,9^

Telemedicine is being used more commonly in neonatology to support health care delivery at medically underserved hospitals that do not have immediate access to in-person neonatologists or pediatricians.^10^ It is a promising method to support the regionalization of neonatal care and has been proven to improve the quality of neonatal resuscitation and reduce infant mortality.^11,12^ In simulated studies, video telemedicine influenced the perceived stability of neonatal patients requiring medical transport.^13^

Telecoaching has been utilized to support other cardiopulmonary resuscitation (CPR) training programs such as Advanced Cardiovascular Life Support and Pediatric Advanced Life Support.^14^ In-person PPV coaching improved PPV performance in experienced neonatal resuscitation providers.^15^ However, little is known of the impact of telecoaching on PPV performance.

## Materials and Methods

This project was approved by the University of Washington Institutional Review Board. A crossover study design was used. Experienced neonatal intensive care unit (NICU) nurses and respiratory therapists were recruited to participate in this pilot study during an NRP course. Demographic information on age, self-identified gender, role, years of experience, time since last NRP training, and volume of newborn resuscitations in the prior 6 months was collected. Participants were randomly assigned to receive the intervention with real-time PPV telecoaching through Zoom video link (Zoom Communications Inc., San Jose, CA) or perform independent PPV without coaching (control) for 30 sec. They were subsequently switched to the opposite modality for another 30-sec period.

Using a self-inflating bag, commonly recommended for use in low-resource environments where telecoaching may be required, each participant was asked to provide effective PPV to a term neonatal manikin using a 250 mL neonatal self-inflating ventilation bag (Ambu A/S^®^, Ballerup, Denmark), and a round face mask (Neonatal Face Mask size 1).^16^ They used the one-handed “EC” technique where the little, ring, and middle fingers form an “E” and rest on the mandible and provide chin lift, whereas the index finger and the thumb form a “C” around the mask to achieve mask seal. A positive end expiratory pressure valve and manometer were attached to the ventilation bag, and the pop-off valve was set to be activated at 40 mmH_2_O. No external gas flow was connected to the ventilation bag.

**FIG. 1.**
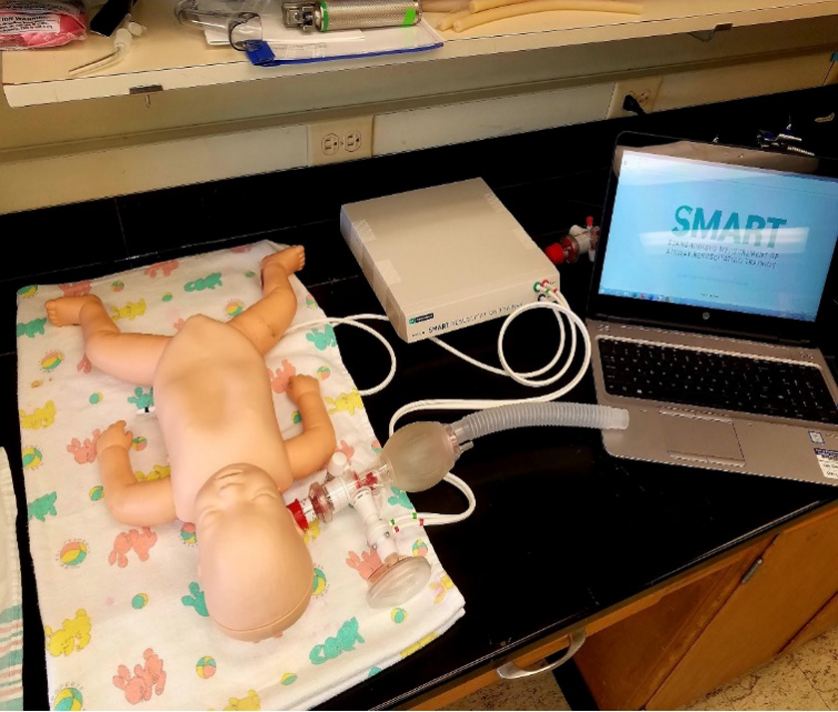
SMART© manikin resuscitation station. SMART, Standardized Measurement of Airway Resuscitation Training.

**FIG. 2.**
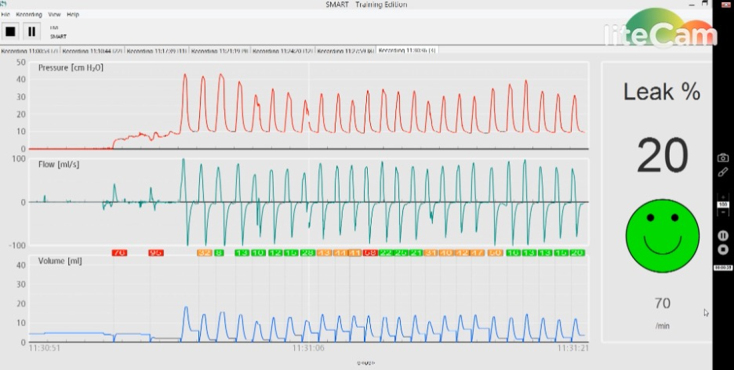
SMART© system data output. cmH_2_O, centimeters of water.

Quality of bag-valve-mask ventilation was assessed using a term neonatal manikin with a respiratory function monitor for training purposes (Standardized Measurement of Airway Resuscitation Training [SMART]©; GM Instruments Ltd., United Kingdom). This consists of a control unit connected to a flow sensor (F10L screen pneumotachograph; GM Instruments Ltd.) and a term neonatal manikin with leak-free airway and an integrated neonatal test lung (Draeger©; Drägerwerk AG & Co. KGaA, Germany; Fig. 1). For each inflation, peak inspiratory pressure, airflow, tidal volume (TV), respiratory rate, and face mask leakage are displayed on a connected personal computer in live time (Fig. 2). The participants and remote coach were unable to see the SMART screen output throughout the assessments.

During the telecoaching session, participants received specific instruction and feedback from the remote instructor who observed their technique through Zoom. The video stream showed the instructor how the participant placed the mask on the manikin's face and how much chest rise was achieved with each inflation. The telecoaching instructions provided were directed around the NRP course mnemonic MR SOPA (Mask replacement, Reposition head, Suction, Open mouth, Increase pressure, and Alternative airway).

If subjects were not achieving chest rise on the manikin, the telecoach provided directed feedback to progress through the MR SOPA steps, including replacing the face mask, repositioning the manikin's head, and squeezing the bag harder if increased pressure was needed. The goal was to achieve visible chest rise, and the peak inspiratory pressure was titrated upward from the standard starting place of 20 cmH_2_O per NRP guidelines until chest rise was satisfactory. Suctioning the airway was not performed as there were no secretions. Opening the mouth further was not possible in the manikin.

Peak inspiratory pressure (PIP), TV, respiratory rate, and mask leak were captured by the SMART system for each breath. The median and interquartile ranges (IQRs) of these variables of each breath delivered were calculated for each 30-sec interval. Electronic screen capture was used to collect live-time data on respiratory rate, and mask leak provided by the SMART system during each round of PPV.

The SMART sensor calculates TVs accurately up to a threshold of 80% mask leakage. TV measurements are derived from air flow through the circuit, whereas pressure is measured directly. The rate was counted manually from the video screen capture. The screen capture videos were saved to a server and de-identified. All videos were later analyzed by a single reviewer who was blinded to whether the subjects received coaching during the PPV attempt (Mark Castera).

Peak inspiratory pressure (PIP), TV, respiratory rate, and mask leak were compared between telecoaching and standard practice sessions using the STATA version 14.2 statistical software program (© STATACorp LLC, College Station, TX) and SigmaStat version 4.0 (© Systat Software, San Jose, CA). Histograms demonstrated that pressure, flow, volume, and mask leak were not normally distributed so data were summarized with medians and IQRs and compared with nonparametric testing through the Wilcoxon rank-sum and signed-rank tests.

Flow and TV measurements could not be compared between groups because the SMART sensor does not reliably measure these variables at >80% mask leak, and the control group's median leak was 100%. Respiratory rates were found to be normally distributed so are presented as means with standard deviations and analyzed with *t*-tests. A *p*-value of <0.05 was considered statistically significant for all comparisons.

## Results

**FIG. 3.**
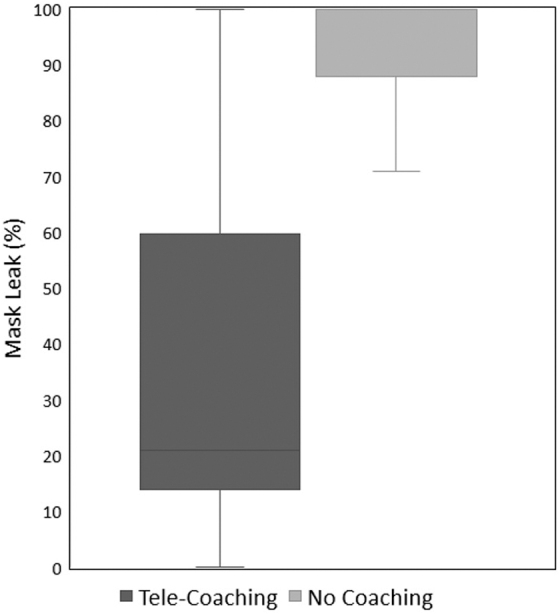
Mask leak percentage.

A total of 14 health professionals (11 nurses and 3 respiratory therapists) with a median of 10.5 years [IQR 5–15.5]) of experience participated in this pilot study (Table 1). More than half of these participants had attended five or fewer deliveries in the past 6 months. Before this day's NRP course, 86% of these providers had not participated in an NRP training course in over 12 months. Figure 3 shows that mask leak was significantly improved during the telecoaching sessions compared with the control sessions (median 19% vs. 100%, *p* = 0.0001, Wilcoxon rank-sum).

**Table 1.** Demographics

Participant demographics (***n*** = 14)	***n*** (%)
How long since last NRP training? months	
6–12	1 (7)
>12	12 (86)
No previous NRP course	1 (7)
Role	
RN	11 (79)
RT	3 (31)
Years of experience	
1–10	7 (50)
11–20	4 (29)
21–30	0 (0)
31–40	3 (21)
Resuscitations in past 6 months	
None	4 (29)
1–5	4 (29)
6–10	4 (29)
11–15	0 (0)
16–20	1 (7)
21–25	0 (0)
>25	1 (7)
Age	
25–35	7 (50)
36–45	2 (14)
46–55	2 (14)
>56	3 (21)
Gender	
Female	12 (86)
Male	2 (14)
Race/ethnicity^a^	
Non-Hispanic white	11 (79)
Hispanic	3 (21)

^a^ No participants self-identified as any other group.

NRP, neonatal resuscitation program; RN, registered nurse; RT, respiratory therapist.

**FIG. 4.**
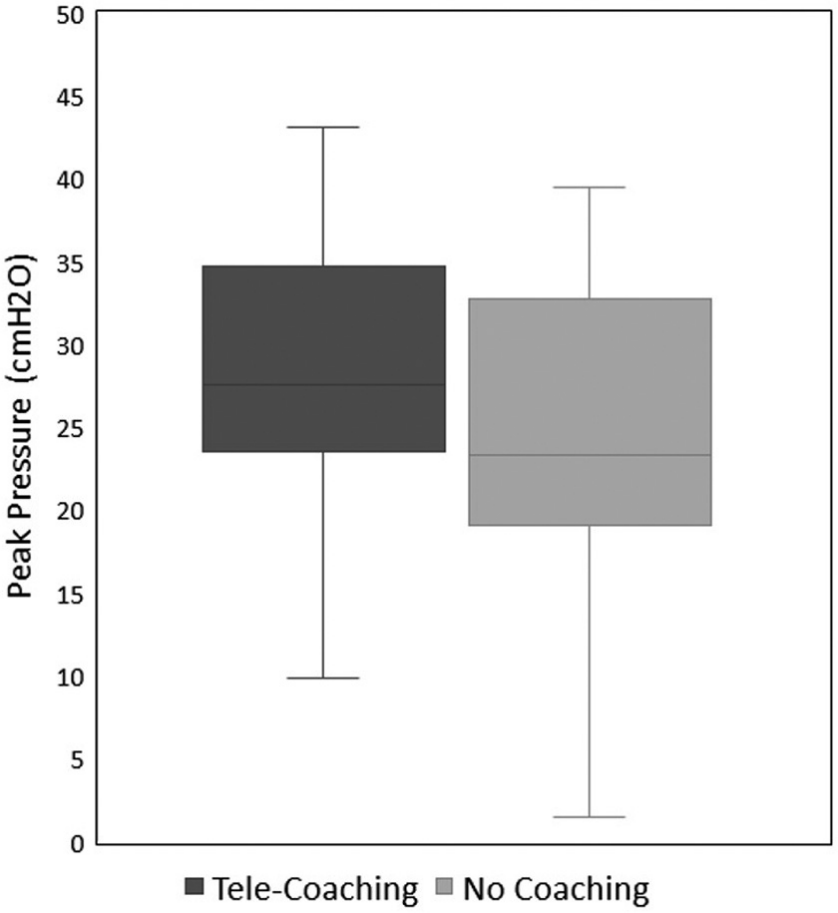
Peak inspiratory pressure delivery during PPV.

Peak inspiratory pressure delivery was higher during telecoaching (median 27.6 [IQR 23.5–34.7] vs. 23.3 [IQR 19.1–32.8] cmH_2_O, *p* < 0.001) as shown in Figure 4. Respiratory rates delivered during the telecoaching and control sessions had no statistically significant difference (55 breaths/min telecoaching vs. 58 breaths/min control, *p* = 0.51, *t*-test).

## Discussion

In this study on the effects of telecoaching during PPV training for neonatal resuscitation providers, we found that telecoaching resulted in less mask leak and higher levels of pressure delivery. We measured substantial improvements in PPV performance in the intervention group through data collected by the SMART mannequin device. Many of our study participants had previous experience in providing neonatal resuscitation care. The audiovisual equipment required to set up this training environment is simple and could be replicated easily in a simulation setting.

In our study, neonatal care providers delivering PPV without telecoaching had very high mask leak, which was improved with the use of telecoaching. Studies on in-person coaching during simulated resuscitation have demonstrated that coaching improved PPV and CPR performance.^15,17^ Our study's mask leak change appears greater than previous in-person PPV coaching that reported less mask leak in the control group using the same SMART mannequin (39% leak with in-person coaching, IQR 21–70 vs. 45% without coaching, IQR 22–98; *p* = 0.005).^15^ Successful resuscitation of apneic newborns relies on the appropriate delivery of PPV. Mask leak can interfere with the delivery of air into the lungs, resulting in failure to provide adequate distending pressure to inflate the newborn lung.^18^

Even when the mask is appropriately sized, it can be difficult to avoid some mask leak during PPV due to the technical difficulty of applying the fixed shape mask to the unique contours of a newborn's face. For this reason, both the NRP and HBB programs place a priority on training health care workers on the appropriate delivery of PPV.^4^ The NRP algorithm guides neonatal resuscitation providers, including ventilation corrective steps, and prior studies suggest that telemedicine supports adherence to the NRP algorithm.^11,19,20^

Within the NRP algorithm, one of the key ventilation corrective steps is adjusting the mask to ensure a proper seal is made to reduce air leak. It is challenging for many NRP providers to accurately self-assess their seal of the infant's mouth and nose with the ventilation mask because PPV devices can maintain pressure with leaks >55%.^21^

Self-inflating bag manometers are a poor proxy for determining mask seal and can be misleading for resuscitation providers.^22^ If the infant does not respond to ventilation corrective steps, the resuscitation provider must assume that the infant is not responding to noninvasive PPV and proceed to endotracheal intubation, a procedure that requires substantial technical skills and carries risks such as oxygen desaturations, esophageal intubation, and cardiac dysrhythmias.^23^

Current statistics show that <1% of newborn infants worldwide require escalations to advanced interventions such as invasive ventilation or chest compressions, but among that 1%, it is unclear which of those infants received truly effective noninvasive PPV.^1^ It is important to improve outcomes for this population of depressed infants and reduce unnecessary endotracheal intubations. Our study shows that telecoaching may be a viable means to enhancing resuscitation providers' PPV performance during simulations.

Our findings suggest that in a simulated setting, participants provided increased pressure to the airway during the telecoaching compared with performing the procedure independently. One of the main goals of providing ventilatory support to neonates with respiratory distress is to improve their functional residual capacity (FRC).^18^ Although there is debate about the approach to lung recruitment in establishing FRC, providing consistent ventilation with adequate pressure with each PPV breath is crucial to the success of resuscitation.^24,25^

The guidance for ventilation corrective steps includes a recommendation to increase pressure if the infant is not responding to initial PPV attempts. If the mask is not applied properly, the increased pressure will not be delivered to the airway, a missed opportunity for recovery before invasive procedures are done.

An important balancing measure to pressure delivery is pneumothorax due to overdistension of the lungs. Telemedicine is increasingly used to support newborn resuscitation. In one retrospective review, newborns that received telemedicine-supported resuscitation had more cases of pneumothoraxes (11% vs. 2%).^19^ This may be related to reduced mask leak from remote coaching resulting in higher TVs or visual limitations of the remote coach.

The remote coach relies on visualizing chest rise as an indicator of adequate pressure delivery and might recommend increasing pressure inappropriately if the video lacks clarity. In our study, measured PIP's in the telecoaching group were higher than desired. This may have been a result of decreased mask leak, poor visualization of chest rise in the manikin, or a combination of the two.

Teams carrying out CPRs of an older child or adult often designate an individual to act as a CPR coach for team members performing chest compressions. This can provide helpful real-time feedback and lead to improved outcomes as high-quality chest compressions are closely linked to the survival of a cardiac arrest event.^26^ Promising results have been found in adult models using smartphones for telecoaching for chest compression quality.^14^

Designating an in-person “PPV coach” may be similarly beneficial, but the physical constraints of limited space around a warmed bed may limit feasibility. The task of monitoring PPV quality is often left to the resuscitation leader at the head of the bed who must divide his or her attention between the infant and other variables such as cardiorespiratory monitors or Apgar timers.

Our study offers a potential solution to this challenge with the use of a camera with a live video stream to another team member who could act as a PPV coach remote from the bedside. The use of a self-inflating bag in our study allows for extrapolation to low-resource and transport environments where T-piece resuscitators and/or gas flow may not be available, but in which telemedicine is increasingly being considered feasible and acceptable to support neonatal resuscitation and care.^16,27^ Future studies could explore the feasibility and outcomes of such a remote PPV coaching model.

No significant difference was noted in ventilatory rates between the groups with both groups generally providing between 50 and 60 breaths per minute. This is within the goal range of 40–60 breaths per minute recommended by current NRP guidelines.^28^ The ability of these participants to provide PPV at the recommended rate was consistent with other studies that note that errors in ventilatory rate are relatively uncommon in NRP trained providers.^2^ The use of tools such as metronomes has been found to improve the accuracy of delivered ventilation and chest compression rates in CPR.^29,30^

In the delivery of PPV, NRP recommends that providers use counting methods such as “breathe, 2, 3” to increase the accuracy of manually delivered ventilation rates.^28^ For experienced learners, the use of these tools may help to maintain awareness of their ventilatory rates. This is particularly true when the PPV provider does not have other roles as in our study. However, if the PPV provider has additional roles, such as that of team leader, then cognitive load and loss of situational awareness may result in the need for additional support through remote or in-person coaching to maintain appropriate ventilatory rates.^31^

This study has some limitations. This was a pilot study at a single NICU with a small sample size. Although we included experienced respiratory therapists and nurses, team members who often take the role of PPV provider, other NICU team members may also be involved in neonatal resuscitations. The SMART mannequin has limits in its accuracy at high mask leak percentages that limited analysis. TV measurement has important clinical implications, but it had to be omitted from data analysis due to erratic measurements during times of extremely high mask leak.

It is unclear why participants providing PPV independently (control state) demonstrate such profoundly high mask leak on the mannequin despite having just completed an NRP refresher course. The SMART mannequin may have a differently shaped face than the mannequin used during the NRP course, which could present difficulty in adapting their technique initially. Future study is needed to confirm this high rate of mask leak and address it during NRP training to prevent clinical harm from inadequate PPV.

## Conclusion

Participants demonstrated better PPV performance during telecoaching with lower leak percentages. They also achieved higher peak inspiratory pressure delivery to the airway. This study demonstrates the feasibility of providing real-time feedback to NRP providers through video connection by a remote facilitator. Real-time PPV coaching may support the delivery of effective PPV during neonatal resuscitation simulations. Future studies should investigate the ability of telecoaching to improve PPV delivery and support neonatal resuscitation performance in clinical settings.

## Abbreviations Used

CPR: cardiopulmonary resuscitation
FRC: functional residual capacity
HBB: Helping Babies Breathe
IQR: interquartile range
NICU: neonatal intensive care unit
NRP: neonatal resuscitation program
PIP: peak inspiratory pressure
PPV: positive pressure ventilation
SMART: Standardized Measurement of Airway Resuscitation Training
TV: tidal volume
